# Distinct Molecular Mechanisms of Host Immune Response Modulation by Arenavirus NP and Z Proteins

**DOI:** 10.3390/v12070784

**Published:** 2020-07-21

**Authors:** Robert J. Stott, Thomas Strecker, Toshana L. Foster

**Affiliations:** 1 Faculty of Medicine and Health Sciences, School of Veterinary Medicine and Science, The University of Nottingham, Sutton Bonington Campus, Loughborough LE12 5RD, UK; robert.stott@nottingham.ac.uk; 2 Institut für Virologie der Philipps-Universität Marburg, Hans-Meerwein-Str. 2, 35043 Marburg, Germany

**Keywords:** arenavirus, Lassa virus, host antiviral response, virus-host interactions, innate immunity, nucleoprotein NP, matrix protein Z, intrinsic immunity, restriction factor

## Abstract

Endemic to West Africa and South America, mammalian arenaviruses can cross the species barrier from their natural rodent hosts to humans, resulting in illnesses ranging from mild flu-like syndromes to severe and fatal haemorrhagic zoonoses. The increased frequency of outbreaks and associated high fatality rates of the most prevalent arenavirus, Lassa, in West African countries, highlights the significant risk to public health and to the socio-economic development of affected countries. The devastating impact of these viruses is further exacerbated by the lack of approved vaccines and effective treatments. Differential immune responses to arenavirus infections that can lead to either clearance or rapid, widespread and uncontrolled viral dissemination are modulated by the arenavirus multifunctional proteins, NP and Z. These two proteins control the antiviral response to infection by targeting multiple cellular pathways; and thus, represent attractive targets for antiviral development to counteract infection. The interplay between the host immune responses and viral replication is a key determinant of virus pathogenicity and disease outcome. In this review, we examine the current understanding of host immune defenses against arenavirus infections and summarise the host protein interactions of NP and Z and the mechanisms that govern immune evasion strategies.

## 1. Introduction

RNA viruses, despite the limited size of their genomes, pose serious challenges to global public health [1,2,3]. A multitude of antiviral host immune mechanisms have evolved to inhibit viral replication processes, from virus entry through to exit from the infected host cell. Virus-host interactions have evolved to enable these viruses to resist or avoid host antiviral responses. These complex interactions are governed by the limited number, but multifunctional proteins that are encoded by these small viruses [4,5].

Public health concerns have heightened due to the increased incidence of arenavirus epidemics in endemic countries; this is worsened by the lack of vaccines and treatments available. The ongoing, devastating outbreaks of Lassa virus (LASV), the most prevalent arenavirus endemic to Western Africa, highlight the importance of understanding the nature and intricacy of key interactions that occur during emerging RNA virus infections [6,7,8,9]. Severe disease associated with LASV infection is characterised by general immunosuppression that contributes to high-level viremia and reflects the ability of LASV to counteract host antiviral responses. This hallmark of infection is driven by the evasion strategies of two of the four arenavirus-encoded proteins—the nucleoprotein (NP) and the matrix protein (Z) [10]. An overview of the differential mechanisms of host immune modulation by NP and Z proteins of pathogenic and non-pathogenic arenaviruses that influence disease outcomes, is given below. A better understanding of these mechanisms will define important host countermeasures and will aid in the development of novel, effective therapeutic approaches. 

### The Arenaviridae Family and Relevance to Human Health

**Figure 1:**
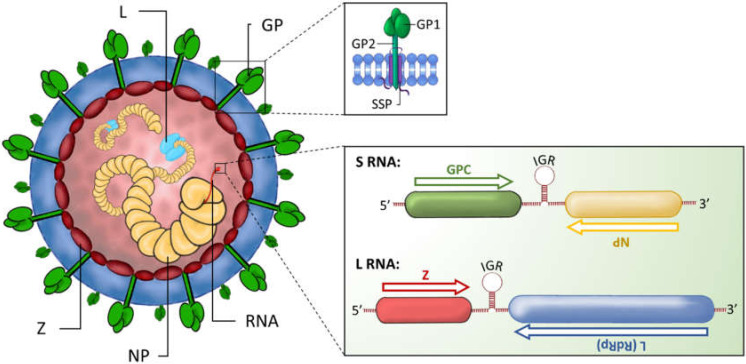
Mammarenavirus structure and genome composition. Mammarenaviruses have a spherical, enveloped structure. The outer surface contains the glycoprotein spike complex GP consisting of the subunits GP1, GP2 and SSP (inset) involved in receptor binding and host cell entry. Within the enveloped structure the small zinc finger matrix protein (Z) encloses the nucleocapsid which is formed of the nucleoprotein (NP) surrounding the small (S) and large (L) RNA segments, and the RNA-dependent RNA polymerase (RdRp) L protein. Mammarenaviruses possess a bi-segmented single-stranded RNA genome. Both RNA segments comprise two open reading frames (ORFs) that are separated by non-coding intergenic regions (IGR) and involved in RNA transcription termination. The S RNA segment encodes the glycoprotein precursor (GPC) and the NP, whereas the L RNA segment encodes the Z protein and the L RNA-dependent RNA polymerase.

The *Arenaviridae* family is a large group of diverse, enveloped, negative-sense RNA viruses. Members of this family are classified into four distinct genera: *Antennavirus, Mammarenavirus, Hartmanivirus*, and *Reptarenavirus*, based on their natural reservoir hosts—fish, rodents, and reptiles for both *Hartmanivirus* and *Reptarenavirus*, respectively [11,12,13]. Other than the tri-segmented *Antennavirus* genus, members of the *Arenaviridae* family possess a bi-segmented, single-stranded RNA genome consisting of one large (L) segment (~7.2 kb) and one small (S) segment (~3.5 kb). Each segment comprises two open reading frames (ORFs) that encode for two non-overlapping gene products in an ambisense orientation. Termination of viral RNA transcription is orchestrated by a highly structured, non-coding intergenic region (IGR) that separates the two ORFs (Figure 1) [14].

The S segment encodes the immature glycoprotein precursor polyprotein GPC that is co- and post-translationally cleaved into GP1 and GP2 and the stable signal peptide (SSP) [15,16,17]. These three protein subunits form the mature glycoprotein spike complex (referred to as GP) on the viral surface. GP1 is involved in receptor binding and entry into host cells, while GP2 and SSP are implicated in stabilising receptor-GP complexes and in viral fusion within host cell membranes [18,19,20]. The S segment also encodes for a major structural component of the nucleocapsid: the nucleoprotein, NP which is abundantly produced during infection.

The L segment encodes for the RNA-dependent RNA polymerase (RdRp) that is responsible for initiating replication of the RNA genome, and also encodes for the small, zinc finger matrix protein, Z, that is involved in regulating viral RNA synthesis, virion assembly and budding (Figure 1) [21,22,23]. Notably, the genome of hartmaniviruses lacks an open reading frame for the Z protein, suggesting that the replication cycle of these viruses is mechanistically distinct from other arenaviruses [13]. Indeed, it is hypothesised that like hantaviruses, GP2 of hartmaniviruses is able to bind to members of the endosomal sorting complex required for transport (ESCRT) pathway to orchestrate virus budding at the plasma membrane. This implies that GP2 may act as a Z protein surrogate, bypassing the need for Z protein expression in hartmaniviruses [13,24].

All human pathogens are members of the large *Mammarenavirus* genus that currently encompasses 39 recognised virus species, as defined by the International Committee on Taxonomy of Viruses. Based on phylogenetic and serological characteristics, as well as geographical prevalence, mammalian arenaviruses are further divided into Old World (OW) and New World (NW) groups, representing viruses endemic to Africa and the Americas, respectively [25,26]. NW arenaviruses are differentiated further into clades A, B, C and A/B (recombinant species otherwise referred to as clade D) [27,28,29,30]. The OW arenaviruses include the globally distributed, prototypic arenavirus lymphocytic choriomeningitis virus (LCMV) that is associated with aseptic meningitis; the highly pathogenic LASV that is associated with severe viral haemorrhagic fever (VHF) with over 5000 reported deaths annually in endemic West African countries; and the recently emerged Lujo virus (LUJV), associated with severe haemorrhagic fever disease in South Africa and Zambia [31]. The NW group of arenaviruses also includes those that cause haemorrhagic fever in humans with associated high fatality rates of between 15% and 33%, namely: Machupo virus (MACV), Junín virus (JUNV), Guanarito virus (GTOV), Sabia virus (SABV) and Chapare virus (CHAPV) [12,32,33,34,35].

The natural hosts for both OW and NW mammarenaviruses are the Muridae family of mice, with the global spread of host habitats governing the geographical distribution of the viruses [30]. OW mammarenaviruses are found in the Murinae sub-family of mice restricted to Africa. The multimammate rat *Mastomys natalensis* and related species (*Hylomyscus pamfi* and *Mastomys erythroleucus*), distributed around West Africa, are the main reservoir hosts of LASV. In contrast, LCMV circulates in the *Mus musculus* host, found globally [36]. The Neotominae and Sigmodontinae sub-families of mice, found in North and South America, are the natural reservoir hosts of NW mammarenaviruses, with the exception of the NW clade B arenavirus Tacaribe virus (TCRV). TCRV appears to be carried by, and cause disease in, Artibeus bats [37,38]. As zoonotic pathogens, mammarenaviruses cause persistent and asymptomatic infections in their natural hosts and transmission to humans usually occurs in rural endemic regions through contact with infectious murine excretions or consumption of rodent meat [39,40]. Host and viral factors determine the variability in disease pathogenesis, varying from control of the viral infection and clearance by host immune responses, to a persistent infection, to severe and often fatal haemorrhagic fever. LASV infection frequently presents as an asymptomatic disease in humans; if symptoms do occur, they appear after an incubation period of around 10 days (range 3–21 days), characteristically as non-specific flu-like symptoms including malaise, weakness, fever and headaches [41]. In severe cases, however, patients with Lassa fever (LF) can develop haemorrhagic manifestations and multi-organ failure with fatal outcome. LF can have fatality rates as high as 25% in healthcare settings where nosocomial transmission of LASV infections has been reported, due to exposure to highly viraemic patients under poor hygiene and sanitation conditions and the lack of proper barrier nursing and infection control [8,9,41]. Human-to-human transmission of NW JUNV and MACV infections related to nosocomial outbreaks has also been reported [18].

Early diagnosis of arenavirus infection in endemic regions is critical but lacking, as symptoms are similar to other endemic diseases such as malaria, typhoid fever and other VHFs, like Ebola virus disease [41]. An approved antiviral treatment does not exist, but evidence suggests that LASV infection partially responds to treatment with the nucleoside analogue ribavirin, particularly during the early stages of infection [42,43,44]. Administration of ribavirin, however, does not significantly impact on fatality rates when compared to untreated patients, therefore there is an urgent need for improved and effective antivirals [45]. These limited treatment options highlight the urgent need for continued research into developing antiviral therapeutics to target arenavirus infection. This is further exacerbated by the lack of approved vaccines against arenavirus VHFs. The live-attenuated Candid#1 strain of JUNV has shown high efficacy against Argentine haemorrhagic fever caused by the etiological agent, JUNV [34,46]. This vaccine, however, is not licensed outside of Argentina and has been shown to be ineffective against other arenavirus infections, including MACV and LASV. In 2019, the Coalition for Epidemic Preparedness Innovations (CEPI) accelerated two promising LASV vaccine candidates to the phase 1 clinical trial stage, with data of the trials currently pending. The safety and efficacy evaluations of both the preventative LASV DNA vaccine candidate INO-4500 (NCT04093076) and the live attenuated measles (Schwartz strain) virus-based vector expressing GPC and NP (MeV-LASV (NCT04055454)) are the first steps towards developing a comprehensive vaccination program against LASV [47,48,49].

## 2. Host Immune Responses during Arenavirus Infection

**Figure 2:**
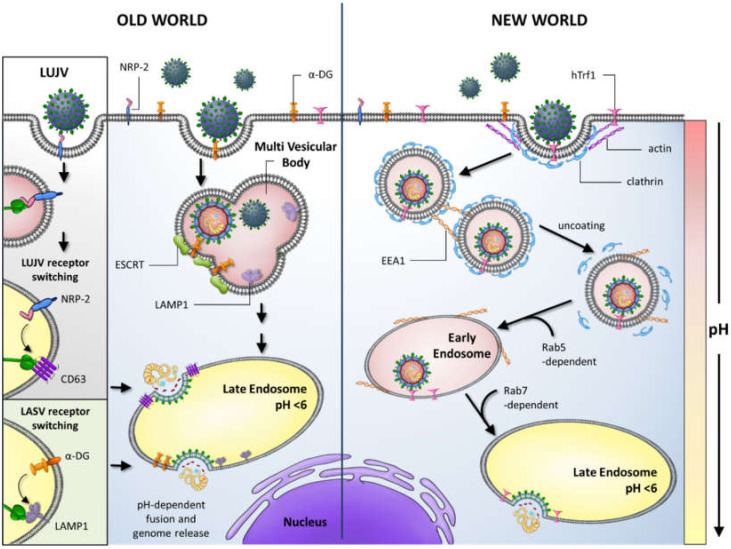
Entry mechanisms of Old World (OW) and New World (NW) arenaviruses. OW arenaviruses bind to α-dystroglycan (α-DG) with the exception of Lujo virus (LUJV) which binds NRP-2. OW arenaviruses enter via a clathrin-independent mechanism involving multivesicular body formation and sorting through the endosomal sorting complex required for transport (ESCRT) pathway. Highly pathogenic NW arenaviruses use human transferrin receptor 1 (hTrf1), or species-specific orthologs, and enter via clathrin-mediated mechanisms. NW virus particles are delivered to EEA1-positive endosomes and then to late endosomal compartments in a Rab5 and Rab7-dependent manner. Upon exposure to low pH in the late endosome, conformational changes in the arenavirus glycoprotein lead to fusion and release of the viral genome into the cytoplasm. Lassa virus (LASV) particles require a receptor switch to lysosome associated membrane protein 1 (LAMP1) in the late endosome. Similarly, LUJV switches to CD63 to mediate fusion.

A major barrier to zoonotic virus infection is the recognition of a suitable cell surface receptor on susceptible human cells. The GP1 subunit of several OW arenaviruses, including LASV and LCMV, and Clade C NW arenaviruses, binds to the widely cell surface-expressed α-dystroglycan (α-DG) receptor (Figure 2). 

Fusion following endocytosis of LASV particles requires a GP conformation and pH-triggered receptor switch to the endosomal cellular protein lysosome associated membrane protein 1 (LAMP1) [50,51]. Similarly, OW LUJV adopts an analogous two-step mechanism for entry by binding to the cell surface molecule NRP-2 (neuropilin-2) and switching to the tetraspanin CD63 in endosomal compartments, to mediate fusion with cellular membranes (Figure 2) [52]. Human pathogenic Clade B NW arenaviruses utilise the human transferrin receptor (hTrf1) for entry, whilst non-pathogenic Clade B viruses engage hTrf1 orthologs for entry [53,54]. Following receptor engagement, NW virus particles fuse with cellular membranes via clathrin-mediated mechanisms and are delivered to EEA1-positive endosomes before moving to late endosomal compartments in a Rab5 and Rab7-dependent manner [55]. Fusion for α-DG-dependent viruses, in contrast, occurs via a clathrin-independent mechanism involving multivesicular body formation and sorting through the endosomal sorting complex required for transport (ESCRT) pathway (Figure 2) [55,56,57].

Following fusion, viral genome replication and transcription that results in 5′-capped, non-polyadenylated messenger RNAs (mRNA) encoding the viral proteins, occurs in the cell cytoplasm (Figure 2). Here, arenaviruses encounter the next crucial barrier: the host cell’s innate immune response. This is the initial, non-specific defense system against pathogen invasion that is induced prior to the activation and regulation of the adaptive immune response involving pathogen-specific antibody production and activity of cytotoxic T-lymphocyte (CTL) responses. Viral infection commonly produces pathogen-associated molecular patterns (PAMPs) [58,59]. These unique molecular patterns, such as double stranded RNA (dsRNA) or 5′-triphosphorylated RNA generated during viral RNA replication, are readily recognised by immune cells [60]. Arenavirus RNAs possess 5′-triphosphate containing panhandle structures that comprise the 5′ and 3′ ends of the genomic RNAs as well as the structured IGRs. These PAMPs can be sensed by host pattern recognition receptors (PRRs), including retinoic acid inducible gene 1 (RIG-I)-like receptors (RLRs), Toll-like receptors (TLRs) and protein kinase R (PKR). These PRRs then activate downstream signalling pathways that stimulate an antiviral response in the form of the upregulation or expression of type I interferons (IFN1- IFNα and IFNβ), cytokines, proapoptotic factors and the activation and maturation of innate immune cellular arms such as dendritic cells (DCs), T cells and macrophages [61]. Key to counteraction of viral infection is the IFN signalling pathway that is activated upon binding of secreted IFN1 to target cells. This activation cascade leads to the upregulation or expression of multiple interferon stimulatory genes (ISGs). ISGs can act on the host or more specifically at numerous stages of the viral life cycle, to inhibit virus growth. Intriguingly, host immune responses to different pathogenic and non-pathogenic arenavirus infections, have varying implications on viral pathogenesis and thus therapeutic development. The innate immune response to OW arenavirus infection has been extensively studied during infection with the prototypic member LCMV (reviewed in [62]). LCMV infection leads to either an acute infection which is rapidly cleared, or a persistent infection which causes more severe disease in mouse models. The clearance of acute infection is largely due to the robustness of the IFN response early during infection that is observed to surge around 6–48 h post-infection, usually 3–5 days prior to a peak in viral titer [63,64]. The more substantial an IFN response is in the early stages of infection, the more likely it is that virus-specific CD8+ cytotoxic T cells will be induced and virus clearance will occur, whereas a weaker IFN response leads to the observed chronic and persistent LCMV infections in mice. Target cells for LASV include monocyte-derived DCs (moDCs) and macrophages, but infection does not induce IFN or cytokine responses in these cells [65]. Thus, severe cases of Lassa fever in humans is characteristically associated with low levels of type I interferons and proinflammatory cytokines such as tumor necrosis factor alpha (TNF-α) and a lack of neutralizing antibody presence, given to the subsequent deficiency in stimulation of T cells, DCs and macrophages [66]. Interestingly, non-pathogenic OW Mopeia virus (MOPV), a close genetic relative to LASV, induces a strong initial IFN and cytokine response in infected moDCs and macrophages leading to a sustained T cell activation and immune response [67,68]. Pathogenic NW JUNV and MACV infections induce robust levels of IFN1 and cytokines and unlike LASV do not suppress the innate immune mechanisms but shift towards a proinflammatory response involving an upregulation of IFN-α and TNF-α. These high cytokine and IFN1 levels are proposed to correlate with the severity of haemorrhagic disease. These differential responses are triggered by NP and Z protein-mediated defense mechanisms as summarised in this review.

### Restriction Factors Against Arenavirus Infections

Many ISGs or host restriction factors, complementary to systemic innate and adaptive immune proteins, represent host cellular proteins that interfere with specific steps of the viral life cycle to inhibit infection. Many of these factors are interferon inducible and can potently block viral spread [69,70,71,72,73]. Few, briefly described here, have recently been shown to limit arenavirus entry and exit processes.

Virus entry is a key determinant of viral host range, cellular tropism and disease outcome, hence, targeting this step of the arenavirus life cycle could have significant impact on the control of viral infection. Gamma-interferon-inducible lysosomal thiol reductase (GILT) is a soluble thiol reductase that is highly expressed in the endosome and lysosomal compartments of arenavirus target cells such as epithelial cells, DCs, macrophages; and is IFN-γ inducible in other cell types [74,75]. GILT has been implicated to play a role in the processing of endocytosed immune signatures such as viral glycoproteins and in reducing the acidic nature of the lysosome to facilitate proteolysis [76,77]. Using LASV GP pseudotyped lentiviral particles, Chen and colleagues demonstrated that GILT suppressed the lysosomal entry pathways of LASV; postulating that its thiol reductase activity in lysosomes is required for the restriction, ultimately blocking viral fusion and genome release [78].

**Figure 3:**
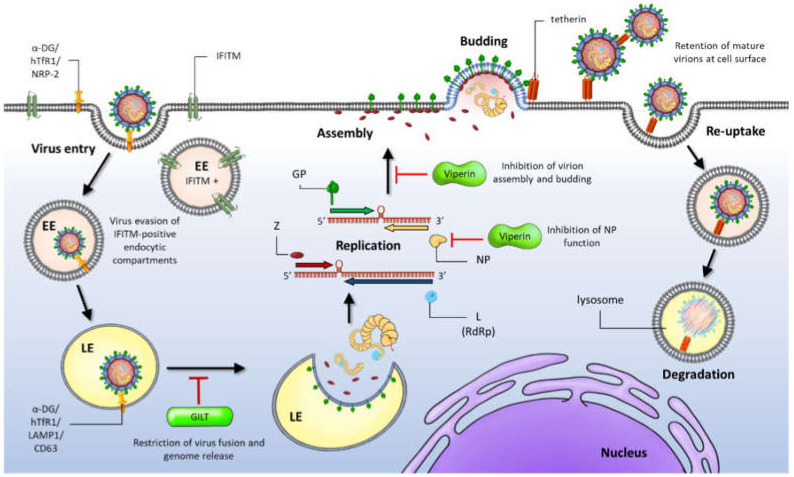
Host restriction factors involved in arenavirus infection. The IFITMs block entry at the sites of fusion within endosomal compartments or at the plasma membrane. Arenavirus particles, however, enter by trafficking through endosomal compartments that lack IFITM expression. Fusion in late endosomal compartments is inhibited by gamma-interferon-inducible lysosomal thiol reductase (GILT) expression. Viperin inhibits virion assembly by restricting the trafficking on arenavirus glycoproteins to the cell surface. Viperin also inhibits NP function by restricting recruitment of replication-transcription complexes orchestrated by NP, to lipid droplets. The membrane protein tetherin mediates retention of budding virions at the cell surface which are then proposed to be re-internalized and delivered to lysosomes for degradation.

One family of ISGs has raised several questions about the entry mechanisms of arenaviruses. The interferon-induced transmembrane (IFITM) family of proteins display broad antiviral activity against the endocytic fusion of enveloped viruses within target cells [71,79,80]. In humans, expression of IFITMs 1, 2 and 3 are potently induced by IFN1 in most cell types but the precise mechanism by which IFITMs restrict virus entry is unclear. Existing explanations include direct modification of membrane content, structure, rigidity and curvature and indirect modification by altering the function of other membrane host proteins, for example the zinc metalloprotease, ZMPSTE24. These proteins are thought to block entry at the sites of fusion within endosomal compartments or at the plasma membrane [80,81]. Using arenavirus-GP pseudotyped particles, trafficking and fusion of these particles was observed to occur in endosomes that lack IFITM expression (Figure 3).

Two independent studies by Suddala et al. and Spence, et al. reported that trafficking of LASV-GP pseudotyped particles appeared to bypass endosomes positive for IFITM3. However, analysis of LASV GP-mediated cell-to-cell fusion using an assay that rely on exposure to low pH, thus avoiding the need for endosomal trafficking, demonstrated that all three IFITMs when expressed in target cells, limited LASV GP pseudotyped fusion [82,83]. This evidence suggests that, instead of being resistant to direct restriction by IFITMs (particularly IFITM3), arenaviruses may employ an avoidance mechanism and use alternative endocytic pathways during entry (Figure 3) [83].

Towards the end of the viral life cycle, virus assembly and budding have been shown to be targeted by the ISGs, viperin and tetherin, respectively (Figure 3). Viperin (virus inhibitory protein, endoplasmic reticulum associated, interferon inducible) is a conserved endoplasmic reticulum protein with an amphipathic α-helix domain at its N-terminus which serves as an anchor to lipid membranes, and was proposed to inhibit JUNV assembly and budding [84]. Using an attenuated strain of JUNV (IV4454), Peña Cárcamo and colleagues showed an induction of viperin expression in JUNV replicating cells. Furthermore, overexpression of viperin reduced efficient virus particle production. The authors proposed that the observed viral glycoprotein mislocalisation in these cells due to lipid raft disruption accounts for the antiviral activity of viperin. Further, it was suggested that a viperin-NP interaction, shown by immunofluorescence and immunoprecipitation experiments, inhibits the recruitment of replication-transcription complexes (RTC) to lipid droplets which is orchestrated by NP, thus inhibiting the function of NP in these processes (Figure 3).

Tetherin (also named bone marrow stromal cell antigen 2 (BST-2) and CD317) is a type II membrane protein inducible by both type I and type II IFNs that is constitutively expressed by plasmacytoid DCs and is expressed by activated T cells [73,85,86,87,88]. Restriction of the physical release of mature virus progeny from infected cells for a broad spectrum of enveloped viruses by tetherin has been well studied and more recently, LASV, MACV and JUNV have been included in this list. The matrix protein Z forms virus-like particles (VLPs) that bud from Z-expressing cells in the absence of other viral proteins, thus several studies have utilised this intrinsic property to study virus budding and demonstrate an inhibition of LASV and MACV VLP release from tetherin over-expressing cells [89,90,91,92]. More recently, Zadeh and colleagues used JUNV-Z VLPs to show an inhibition of Z-mediated particle release by tetherin and showed that propagation of the Candid#1 vaccine strain of JUNV was susceptible to tetherin expression [92]. Interestingly, JUNV infection caused an upregulation of tetherin expression in cells that correlated with increased IFN levels, and a reduction in cell surface expressed tetherin was also observed. Evidence for retention and clustering of VLPs at the cell surface in the presence of tetherin was visualised by electron microscopy. Furthermore, a plausible role of NP, in an as yet not understood mechanism in antagonising restriction that does not involve the downregulation of cell surface tetherin expression, has been proposed [92]. As tetherin also interacts with the host cell endocytic pathway, it is further hypothesised that virions retained at the cell surface by tetherin activity are subjected to re-uptake and trafficking through the endocytic pathway to lysosomes where they are degraded (Figure 3) [93,94,95].

This is a growing area of research that requires further expansion and understanding of virus-host interactions that limit virus spread, including the identification of other restriction factors that may target replication and translation mechanisms. Unravelling the evolution of NP and Z-mediated countermeasures against these host restriction mechanisms will make significant contributions to our knowledge about the arenavirus life cycle and has the potential to influence the development of therapeutic strategies.

## 3. Immunosuppressive Mechanisms of Mammarenavirus NP Proteins

Pathogenic and non-pathogenic OW and NW mammarenaviruses elicit immune responses that are characterised by either weak or robust IFN1 induction [66,67,68,69,70,71]. Several studies, as detailed below, have identified the mechanisms by which NP proteins interfere with PRR activation and the induction of innate immune signalling that account for these differences in immune response.

**Figure 4:**
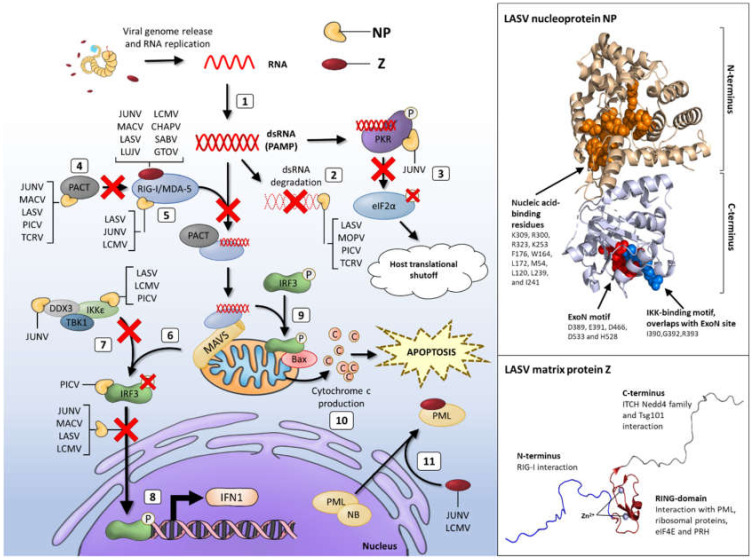
Mechanisms of host immune suppression by arenavirus NP and Z proteins. (1) Arenavirus replication produces pathogen-associated molecular patterns (PAMPs) such as dsRNA which is recognised by pathogen recognition receptors (PRRs) protein kinase R (PKR), retinoic acid inducible gene 1 (RIG-I) or melanoma differentiation-associated protein (MDA)-5. (2) dsRNA is bound and degraded by NP exoribonuclease domain (ExoN) activity (inset- PDB:3MWP). (3) Activated PKR phosphorylates eukaryotic initiation factor 2α (eIF2α) which leads to translational shutdown which is suppressed by JUNV NP ExoN activity. (4) RIG-I sensing of dsRNA is enhanced by the host protein PACT, an interaction also targeted by NP ExoN activity. (5) Direct interaction of NP or Z (inset-PDB:2M1S) proteins with RIG-I and MDA-5 also suppresses PRR signalling. (6) RIG-I associates with mitochondrial antiviral signalling protein (MAVS) and activates interferon responsive factor 3 (IRF3). (7) IRF3 activation is facilitated by DDX3 and IκB kinase ε (IKKε) together with TANK binding kinase 1 (TBK1). DDX3 and IKKε are both suppressed by direct binding of viral NPs. (8) IRF3 induces IFN1 production through translocation to the nucleus, however this translocation is inhibited by arenavirus NPs with PICV directly binding to IRF3 to inhibit activation. (9) RIG-I activation of IRF3 also induces apoptosis through complex formation with the proapoptotic protein Bax which is then translocated to mitochondria. (10) This leads to cytochrome c production and the downstream activation of apoptosis. (11) The RING-domain of arenavirus Z protein has been implicated in abrogating apoptosis induction by relocalising proapoptotic promyelocytic leukemia protein (PML) from the nucleus where it forms PML-nuclear bodies (NBs) to the cytoplasm, here, it targets apoptotic factors such as Bax and caspase activation.

NP is located at the 3′ end of the S RNA segment and is translated from antigenomic sense mRNAs, transcribed directly from viral RNAs, thus along with protein L, is one of the first arenavirus proteins encoded upon infection. NP is the most abundantly expressed protein and orchestrates viral RNA synthesis through binding to viral RNA and thus facilitating transcription and replication [96]. Binding of arenavirus NP to viral genomic RNA is functionally essential to the formation of viral nucleocapsid complexes as well to the suppression of host immune responses (Figure 4, Table 1). 

The nucleocapsid complexes associate with the L protein, mediating viral ribonucleoprotein (vRNP) assembly, and thus replication and transcription. NP also complexes with viral and host proteins to enhance budding and assembly of virions [97]. Biochemical, mutagenesis and structural studies have shown that NP is comprised of an N-terminal RNA-binding domain that mediates vRNP assembly, linked via a flexible C-terminal 3′–5′ exoribonuclease (DEDDh family) domain which is highly conserved across the arenavirus family (Figure 4) [96,98,99,100]. The N-terminal region possesses a unique fold and distinct function as a cap-binding protein that is proposed to bind ssRNA through a gating mechanism of conformational changes. Controversially, this is also suggested to bury the entire m7GpppN mRNA cap structure, with the rest of the mRNA molecule outside of the binding cavity [96,99]. Important to the RNA-binding function of NP is the capacity of this protein to self-associate. Biochemical and mutagenesis assays have shown that NP homo-oligomerisation is required for the replication and transcription functions in forming vRNPs [100,101].

### 3.1. NP-Mediated IFN Inhibition and dsRNA Degradation

The ability of the NP protein to antagonise IFN1 activity has been shown to be conserved by OW LCMV, LASV and NW JUNV, MACV and Pichinde virus (PICV), therefore this inhibitory function is common in arenaviruses that are pathogenic and also non-pathogenic to humans [102]. Mutagenesis studies on LCMV NP by Martínez-Sobrido and colleagues mapped this inhibitory activity to the C-terminal region of NP, identifying amino acid residues 382, 385, and to a lesser extent 386, as critical for antagonising IFN1 induction [103]. Intriguingly, these LCMV mutants did not affect virus RNA replication and the production of infectious virus particles, implying that these functional roles of NP are distinct from its anti-IFN1 activity. These critical residues reside within the highly conserved DIEG NP motif that spans residues 382-385 of OW and NW arenaviruses, including NW TCRV NP. Martínez-Sobrido and colleagues showed that TCRV lacks the ability to counteract the induction of IFN1, with contrastingly high levels of IFN-β and ISG induction observed in TCRV infected cells, despite productive viral replication and release. Given that TCRV NP was observed not to inhibit IFN1-activity, it is likely that other residues outside of the DIEG motif also contribute to the antagonistic function [103]. Indeed, the LCMV C-terminal region spanning 370–553 was found to be crucial for counteracting the IFN1 response and C-terminal deletions of more than 5 residues in the presence of an intact DIEG motif weakened this response [103]. More recent, functional and structural studies on TCRV NP have, however, challenged these previous reports and showed that TCRV NP can inhibit the IFN1 pathway effectively, indicating the importance and conservation of this immunosuppressive activity by arenavirus NPs [104,105].

Subsequent studies observed that this immunosuppressive function of NP is attributed to the C-terminal exonuclease activity of the protein [98]. NP possesses high specificity for dsRNA-specific degradation in the 3′–5′ direction and residues recognised for their role in IFN suppression reside within the exonuclease active site. Studies on non-pathogenic NW PICV virus infection in cell culture and animal models, highlighted the role of the five exoribonuclease catalytic residues needed for IFN1 inhibition, optimal virus propagation and disease pathogenesis. Several studies have demonstrated and postulated that degrading immunostimulatory viral dsRNAs appears to be a common mechanism of arenaviruses, including LASV, LCMV, MOPV, TCRV, and PICV, to evade innate immune responses [99,105,106,107,108]. Residues D389, E391, D466, D533 and H528 (LASV protein numbering) comprise the highly conserved exoribonuclease DEDDh motif (ExoN). These, in addition to proximally located residues including G392, are critical for ExoN activity and virus viability (Figure 4) [109,110]. Some uncertainty exists, however, about the specificity of the ExoN activity amongst the arenavirus family surrounding the degradation of dsRNA. Recent studies by Mateer and colleagues showed that NP ExoN activity of LASV effectively degrades dsRNA, whereas infection with the highly pathogenic NW arenaviruses MACV and JUNV results in a rapid accumulation of dsRNA that is not degraded to a similar degree as in LASV infected cells (Figure 4, Table 1) [110,111]. Mutations of the ExoN function of LASV NP has previously been reported to lead to higher levels of IFN1 in DCs and also in macrophages when compared with wild-type LASV virus infection using a murine polymerase reverse genetics system [109]. Historically, immunofluorescence labelling of dsRNA has been used to visualise the accumulation during RNA virus infection. While this method has been effective during infection with positive-sense RNA viruses with the widely used J2 dsRNA antibody, this has not been sensitive enough for the low level of dsRNA produced during infection with negative-sense RNA viruses [112]. In recent years, the 9D5 monoclonal antibody (MAb) was developed, initially for use in the diagnosis of pan-enterovirus infection [113]. This antibody has been shown to have a high affinity for dsRNA and can detect the dsRNA accumulating during negative-sense RNA virus infection including infection with LCMV and JUNV [111,113]. Thus, with the development of the 9D5 MAb and the use of LASV, JUNV and MACV minigenome replication systems, Mateer et al., [110] were able to visualise dsRNA accumulation. They showed that LASV NP ExoN activity was required for limiting dsRNA accumulation, strengthened by the observation that mutations disrupting LASV ExoN activity led to dsRNA accumulation. Not only did this limitation occur during LASV infection, inhibition of dsRNA accumulation during JUNV infection by co-infection with LASV was measured. Interestingly, expression of LASV NP alone was not sufficient to limit the accumulation of dsRNA in JUNV infected cells, but expression of both LASV NP and L proteins was required to achieve this, suggesting cooperative degradation with viral replication activity (Figure 4, Table 1) [110]. 

The degradation of virus-derived dsRNA may also function to suppress IFN1 expression by preventing PKR signalling and the downstream phosphorylation of eIF2α thus inhibiting host and viral protein translation (Figure 4). Recently, there have been contradicting reports on the ability of JUNV to inhibit phosphorylation of eukaryotic translation initiation factor 2 (eIF2α). A study by King et al. [114] reports that JUNV NP induces phosphorylation of PKR despite not leading to higher levels of phosphorylated eIF2α, whereas other published work suggests that JUNV and MACV infections still result in the phosphorylation of eIF2α [115]. It is important to note that the study by King et al. [114] examined cells infected with the attenuated vaccine strain JUNV Candid#1, whereas Huang et al. [115] used the highly pathogenic JUNV Romero strain. The latter study showed that the NW arenaviruses JUNV and MACV, but not the OW LASV, lead to high levels of activated PKR thus corresponding with the research presented by Mateer et al. [110]. These results highlight the differences observed between the NW JUNV and MACV infections and OW LASV infections in their ability to degrade dsRNA species.

### 3.2. NP Interactions with PRRs

The NP protein of LASV limits the detection of PAMP viral dsRNA to prevent host recognition (Figure 4). Multiple studies corroborate that this recognition is mediated through direct interaction of NP with PRRs, RIG-I and melanoma differentiation-associated protein (MDA)-5, as well as downstream effectors such as IκB kinase ε (IKKε), thereby blocking IRF3 activation [63,102,105,117,124,125]. Upon recognition of viral dsRNA carrying the 5′triphosphate, RIG-I and MDA-5 undergo conformational multimerization and activation that induces production of the early IFN1 species through a signalling cascade, firstly through binding of mitochondrial antiviral signalling protein (MAVS). Formation of these molecular complexes results in the translocation of transcription factors interferon responsive factor (IRF)3 and IRF7 to the nucleus which, along with AP-1 and nuclear factor (NF)-κB, leads to the production of IFN-α, IFN-β and a number of ISGs [126,127]. This signalling cascade usually establishes an antiviral state in neighbouring uninfected cells. This further stimulates immune cell activity such as natural killer (NK) cells, NKT cells, T cells, DCs and macrophages.

In depth investigations using LCMV infection regulation of the IFN1 response, has shown that LCMV NP restricts this response by binding directly to RIG-I and MDA-5 and can block the translocation of IRF3 to the nucleus thus, reducing IFN-β induction [116,128]. Similarly, co-localisation of MACV and JUNV proteins with RIG-I has been observed in JUNV and MACV infected cells implying an upregulation of ISGs indicative of IFN1 activation [110]. Interestingly, this co-localisation was not observed between LASV NP and RIG-I, implying that LASV infection does not activate the IFN-β response in a RIG-I dependent manner. Mutational analysis of residues within the ExoN motif of LCMV and LASV were able to abrogate the inhibitory effect of LCMV NP on IFN1 but did not prevent the binding of NP to RIG-I or MDA-5, demonstrating that the NP-mediated inhibition of IFN1 is only partially due to the interactions with RIG-I and MDA-5 [103,109,128]. A crucial aspect of the induction of IFN1 is the translocation of IRF3 to the nucleus (Figure 4). The NP proteins of both OW and NW arenaviruses including LCMV, LASV, PICV, JUNV and MACV, with the exception of TCRV, can cause a block to both the transcriptional activity and nuclear accumulation of IRF3. The failure of this TCRV NP sequence to inhibit the translocation of IRF3 to the nucleus correlates with the observation of the weaker IFN1 suppression during TCRV infection, reported by Martínez-Sobrido and colleagues, and may contribute to the lack of persistent infection of TCRV observed in rodent species [102,116,119]. The observed inhibition of IRF3 translocation is, however, insufficient to completely block IFN1 expression as demonstrated in LCMV infected mice that still elicit a strong IFN1 response and subsequently a robust host antiviral immune response [129]. Association of LCMV NP with the kinase domain of IKKε, via the exoribonuclease domain (ExoN), has been reported by Pythoud and colleagues, suggesting a sequestration of IKKε which subsequently prevents signalling via the MAVS pathway to induce innate sensors (Figure 4, Table 1) [117].

A 313 amino acid protein known as PACT was shown to enhance RIG-I function through direct interaction [130]. PACT comprises dsRNA-binding motifs (dsRBMs) that bind to dsRNA (dsRBM1 and dsRBM2) and to PKR via dsRBM3 [131]. PACT interacts with the C-terminal repression domain of RIG-I, whilst also activating the ATPase function of RIG-I to potently enhance IFN1 production. PACT potentiates the function of RIG-I at a similar level to that of dsRNA RIG-I ligands. LASV NP has been shown to block the PACT-mediated regulation of RIG-I function, in an ExoN activity-dependent manner [132]. NP does not interfere with the physical interaction between PACT and RIG-I, but as Shao and colleagues infer, NP may degrade dsRNA that is associated with, and essential for the PACT/RIG-I complex function, thereby permitting efficient replication in infected cells in the presence of a dampened immune response (Figure 4, Table 1). This effect could be rescued in the case of a recombinant PICV virus expressing an RNase-defective NP mutation, where virus growth was not measured in wild type mouse embryonic cells [132]. LASV NP function has therefore evolved to counteract stimulation of IFN1 production, through a mechanism that indirectly blocks the activation of RIG-I.

Arenaviruses also activate the innate immune response through the activity of TLRs following the binding of viral PAMPs, thus promoting an IFN1 response [133]. NW JUNV induces activation of a TLR2/TLR6 complex leading to the activation of transcription factors AP-1 and NF-κB and initiation of innate and adaptive responses [134,135,136,137,138,139]. Only TLR2 has been implicated to play a role in OW arenavirus pathogenesis and studies by Hayes et al. have postulated that the observed immunosuppressive phenotype and low level proinflammatory cytokines associated with OW LASV infection is due to a suppression of TLR2- dependent induction of cytokine responses [136].

In addition to the RLRs and TLRs, accumulated dsRNA during NW JUNV, JUNV Candid#1 vaccine strain and MACV infections also activates the ubiquitously expressed host dsRNA sensor, PKR. In infected cells, JUNV NP has been shown to interact with PKR suggesting a localised inhibition of PKR activity to enhance viral protein synthesis [114,115]. Upon dsRNA interaction and binding, PKR undergoes autophosphorylation. Enzymatically activated PKR then initiates the downstream phosphorylation of eIF2α, thus inhibiting the translation of cellular and viral-expressing mRNAs in infected cells [140,141,142]. PKR enhances IFN1 production by stabilising IFN1 mRNA and also activates the transcription factor NF-κB through phosphorylation of IκB [143,144]. Interestingly, JUNV and MACV readily activate PKR seemingly to enhance viral replication through the augmentation of IFN and ISG gene translation, rather than negatively impact viral infection [115]. Similarly, OW LCMV has been shown by King and colleagues to strongly activate PKR but conversely to JUNV, is unable to suppress the kinase activity of PKR, given that transient eIF2α phosphorylation was observed during LCMV infection. This suggests that LCMV may possess alternative mechanisms to inhibit PKR activity or reflects the instability of PKR control over LCMV infection [114]. In contrast, OW LASV infection does not regulate or stimulate PKR activation. This pathway is instead evaded by an unknown mechanism [115]. Therefore, LASV, unlike other arenaviruses, successfully avoids detection by PKR. (Figure 4, Table 1).

### 3.3. NP-DDX3 Interaction Suppresses IFN1 Induction

In recent years, large scale proteomics studies have aimed to provide a global picture of NP host protein interactions [114,145,146]. Through this work, the DEAD (Asp-Glu-Ala-Asp)-box ATP-dependent RNA helicase, DDX3 has been identified as a host interaction partner. DDX3 is thought to play a complex role in host antiviral immunity, acting as a transcriptional regulator of IFN-β promoter and as a viral sensor by interacting with RIG-I and MAVS and a downstream signal transducer of TANK-binding kinase I (TBK1) and IKKε [147,148,149,150]. Interestingly, reports indicate that DDX3 regulates the expression of PACT, thereby inferring a possible strategy that viruses use to counteract antiviral innate immunity [151]. Loureiro and colleagues identified DDX3 as an LCMV NP partner by mass spectrometry of immunoprecipitates from LCMV NP overexpressing cells [146]. Subsequent infection of DDX3 knockout cell lines with LCMV or LASV resulted in reduced virus propagation; an activity that occurred early in infection, independently of IFN1. Mutagenesis studies revealed that both the ATPase and helicase domains are implicated in this role. An extension to the role of DDX3 in suppressing IFN1 production may occur late in LCMV infection, where DDX3 abrogates IFN-β transcription. Interactions of the NP of NW JUNV, MACV and TCRV with DDX3 have been confirmed by immunoprecipitation assays or mass spectrometry analysis, with Loureiro and colleagues demonstrating that like OW arenaviruses, JUNV virus growth required DDX3 expression [114,146]. NP may therefore promote viral spread by sequestering DDX3 from macromolecular complexes, including IKKε, RIG-I or MAVS, that promote IFN1 synthesis [146].

### 3.4. NP-Driven Inhibition of Apoptosis

In addition to the stimulation of the innate immune response upon viral infection, cells can also induce their own death in response to cell stress and damage caused by pathogen invasion, through a mechanism of programmed cell death, known as apoptosis [63]. This effectively limits the spread of pathogens and aids in virus clearance from the host. In order to establish an effective and persistent infection, it is critical, therefore, for viruses to evolve mechanisms of evading the apoptotic pathway or even by using some of the components of this pathway to their benefit [63]. Notably, highly pathogenic JUNV, LASV and LCMV circumvent the induction of apoptosis throughout infection whereas the less pathogenic TCRV and the JUNV Candid#1 vaccine strain induce a robust, caspase-dependent apoptosis response. It is important, however, to note that the growth kinetics of TCRV are similar to those of JUNV, suggesting that induction of apoptosis does not negatively affect TCRV proliferation [65,118,152,153]. The induction of apoptosis by JUNV Candid#1 and TCRV has further been characterised to occur in a RIG-I-dependent and IFN1-independent manner in vitro [154,155]. While the IFN1 response requires IRF3 to translocate to the nucleus, RIG-I-dependent induction of apoptosis activates IRF3 via MAVS and causes IRF3 to interact with the proapoptotic protein Bax. This complex is then transported in the mitochondria which then releases cytochrome c and leads to autocatalytic cleavage of caspase-9 and caspase-3 (Figure 4) [156,157]. An additional function of the arenavirus protein NP has been hypothesised in relation to the induction of apoptosis in infected cells. It has been observed that the NP proteins of several arenaviruses exist in a number of truncated forms [118,152,158]. However, it is not known how, or indeed if these truncations are required for virus replication, assembly or budding. Instead it has been suggested that these truncations are used as a mechanism of suppressing the induction of apoptosis in infected cells. Cleavage of caspases is required for completion of the apoptosis pathway; in JUNV infected cells treated with pan-caspase inhibitors, it was observed that formation of the truncated forms of NP was abrogated [152]. Further, expression of JUNV NP alone by transfection was sufficient to induce caspase cleavage of NP in a similar manner and led to the identification of several caspase cleavage target site motifs. Mutation of these motifs resulted in loss of the truncated forms of JUNV NP and consequently increased levels of apoptosis [152]. These findings indicate a decoy function of arenavirus NP as a substrate for caspase cleavage which inhibits induction of apoptosis and aids in virus dissemination. This decoy substrate hypothesis requires further investigation in the case of other arenaviruses to confirm to what extent this may be a universal strategy of apoptosis suppression by the highly pathogenic arenaviruses. Indeed, it has been reported that other arenaviruses including LASV and PICV produce truncated forms of the NP protein whereas TCRV does not [152,154,159,160,161]. This correlated with the observation that the highly pathogenic JUNV and LASV inhibit apoptosis induction while TCRV infection induces a strong caspase-dependent apoptosis response, that yet, does not impair virus replication. Hence, TCRV appears to lack an NP-mediated anti-apoptotic function and is likely able to modulate apoptosis using diverse strategies [65,118,152,153]. Complementary work by Wolff and colleagues showed that TCRV replication and transcription can induce caspase-dependent apoptosis that does not limit virus growth but is regulated by Z protein expression [153]. This implies that TCRV may exploit the apoptosis pathway in order to enhance virus replication and release, and possibly to evade host immune responses (Section 4) [153].

## 4. Evasion Strategies of the Arenavirus Matrix Protein, Z

The zinc-finger matrix protein Z is the smallest gene product encoded by the L segment of the arenavirus genome. Z proteins of OW and NW arenaviruses possess an N-terminal myristoylation site for insertion into the cell membrane, a central zinc-binding RING finger protein motif and C-terminal late-domain motifs essential for interactions with the cellular ESCRT machinery to facilitate virus budding (Figure 4) [32,162,163]. The NMR structure of the Z protein monomer highlights the intrinsically flexible N- and C-terminal domains that flank the RING domain that binds two co-ordinated zinc atoms. This intrinsic conformational flexibility of the small matrix protein Z may play a significant role in the ability of this protein to adapt to its roles in viral assembly, immune evasion and the regulation of replication and transcription through interaction with various host and viral partners [162,164,165]. The most recent structure of protein Z shows that it can be crystallised in a dodecameric form. Stabilisation of monomeric LASV Z by mutagenesis enhances the negative regulation of replication. In contrast, stabilising the oligomeric, dodecamer state impairs the negative regulatory function, implying that the monomeric and oligomeric forms have differing and possibly opposing functions [162,166]. It will be interesting to further elucidate the molecular details of how protein interactions are modulated by these structural changes to maintain viral replication. [162].

The N-terminal myristoylation is highly conserved amongst the OW and NW arenavirus Z proteins, hence, Z is strongly membrane associated and in the absence of other arenavirus proteins, can form and release enveloped VLPs from the cell surface. Through the recruitment of NP within ribonucleoprotein complexes present and enriched at GP1 and GP2 patches at the cell surface, Z drives the assembly of mature virus progeny [167]. The RING finger protein motif of Z is involved in various protein-protein interactions important for regulating multiple stages of the virus life cycle. LCMV Z was shown to interact with promyelocytic leukaemia protein (PML), a regulator of cell growth, leading to its redistribution from nuclear bodies (NBs) to cytoplasmic bodies, where PML is involved in a number of apoptosis regulating pathways, implying a role in abrogating apoptosis induced upon infection (Figure 4) [158]. Further, PML and Z bind to ribosomal P proteins (P0, P1 and P2) in the nucleus thereby implying a role in regulating protein translation [168,169]. Further, the LCMV PML-Z interaction has been proposed to affect virus production in mouse embryonic fibroblasts inferring an impairment in transcription activity due to the interaction with PML [170]. Interestingly, PML is an IFN-induced protein and has been implicated to mediate anti-viral mechanisms against influenza virus replication amongst other virus families that involves the regulation of the IFN1 promoter and the interferon stimulated response element (ISRE) [158,170]. The accumulation of TCRV Z protein during infection enhances the induction of apoptosis, hence it could be speculated that TCRV utilises this pathway to facilitate replication through a mechanism that involves PML-TCRV Z interactions. Therefore, during TCRV induced apoptosis, redistribution of PML from NBs to the cytoplasm driven by TCRV Z could counteract the induction of IFN1 responses; a mechanism linked to the lack of impaired virus replication observed [153]. Campbell Dwyer and colleagues were able to demonstrate that LCMV Z also interacts with the eukaryotic translation initiation factor 4E (eIF4E) that is crucial for mRNA nuclear cytoplasmic transport, for assembly of transcripts onto polysomes and for initiation of translation. By binding to eIF4E, Z is able to suppress protein production at the post-transcriptional and post-RNA transport level. Therefore, preferential translation of viral transcripts over cellular mRNA in a self-regulating mechanism that occurs later in the infection process may be linked to the viral persistence in chronic infections [171,172]. The proline-rich homeodomain (PRH), a cellular transcription factor that regulates the development of the brain, thyroid and liver, also associates with the Z protein RING domain. Binding of the Z protein of pathogenic and non-pathogenic strains of LCMV with PRH has been observed, but PRH-downregulation by pathogenic LCMV alone in human hepatic cell lines implies that Z suppresses the antiproliferative effects of PRH, hence stimulating cell division that is supportive of viral replication and disease pathogenesis in the absence of liver cell regeneration [173]. Whether other viral and host proteins are involved in this downregulation of PRH remains unclear.

The C-terminal portion of Z contains small conserved tetrapeptide (P[T/S]AP- and/or PPxY-type) motifs, known as late domains, that drive virus particle release through the recruitment of ESCRT proteins that result in the final cellular membrane scission stage required for budding [23,32,174,175]. While the important role of the ESCRT machinery and ESCRT-associated host factors in arenavirus budding is well documented [21,176,177,178], the exact molecular mechanism underlying ESCRT protein recruitment and function to promote virus release remains to be fully elucidated. For example, the P[T/S]AP and PPxY motifs vary substantially both in their number and combination between different Z species, suggesting that arenaviruses have evolved different strategies to gain access to the ESCRT pathway [32]. In addition, both OW and NW Z proteins possess a conserved YxxL-type late domain located within the central RING domain. In the case of NW TCRV and OW MOPV, the YxxL motif does not contribute to the self-budding activity of Z, but it is critical for NP-mediated enhancement of Z-driven VLP budding as well as the incorporation of NP into VLPs [97,177,179]. Using a yeast two-hybrid screen, Baillet and colleagues recently uncovered the interaction of two members of the Nedd4 family of HECT E3 ubiquitin ligases, ITCH and WWP1 with Z proteins of MOPV and LASV [180]. They observed that ITCH was needed for efficient production of infectious virus particles of LCMV, LASV, LUJV and MOPV; and found that direct interaction with this pro-viral host factor was dependent on the PPxY late domain of LASV and MOPV. Thus, this host protein acts as a positive regulator of the late stages of virus infection by enhancing the processes of viral release and virus production [180]. Independently, a proteomics study conducted by Ziegler and colleagues identified Nedd4 family ubiquitin ligases, including ITCH and WWP1, as partners of the LCMV Z protein, binding specifically to the PPxY motif of LCMV Z. They demonstrated that these ligases ubiquitinate LCMV Z, a process that was dispensable for virus release but needed for defective interfering (DI) particle release. These data infer that ubiquitination of other cellular or viral targets than the Z protein by Nedd4 ligases may be the essential link to the ESCRT machinery and enhancement of viral budding [181]. Alongside ubiquitination as a regulator of viral budding, Ziegler and colleagues identified two phosphorylation sites, Y97 and S98 (LASV numbering), located at the C-terminal tail of protein Z and overlapping with the late domain region that could also influence Z protein function. These sites are postulated to play a role in regulating virus budding and opens up new research questions surrounding the host protein binding repertoire of Z protein, given the protein’s conformational flexibility, as well as the corresponding functions related to these new findings [182].

### Z Protein-Mediated Inhibition of IFN Responses

Like NP, the Z protein is also able to regulate the host cell interferon system [183,184]. Sequestration of eIF4E by the Z protein as discussed previously is one such mechanism. This can lead to repression of the production of key host regulators of IFN1 immune responses, such as IRF-7 that is crucial for the enhanced transcription of IFN1 genes, including IFN-β and IFN-α genes [171]. Similar to NP, interaction of arenavirus Z proteins with RIG-I prevents further binding to MAVS and therefore inhibits the production of IFN-β and reduces antiviral host immune responses. Xing and colleagues reported the binding of all Z proteins of pathogenic arenaviruses, including LASV, LCMV, JUNV, CHAPV, SABV, and GTOV to RLRs, via the N-terminal domain, leading to a suppression of IFN1 through inhibition of the RIG-I-MAVS interaction (Figure 4) [184]. This finding was further strengthened by swapping the N-terminal domain of the non-pathogenic PICV with the N-terminal domain of LCMV or LASV Z proteins. Recombinant PICV expressing LCMV or LASV Z protein N-terminal domain was able to bind RIG-I whereas the wild-type PICV was not [184]. Research from the same lab also confirmed this by showing that expression of the LCMV Z as a chimeric protein in the non-pathogenic PICV was sufficient to bind RIG-I and inhibit macrophage activity [185]. Although the inhibition of RIG-I signalling by highly pathogenic arenaviruses in vitro suggests that there should be limited IFN1 response, it is notable that in vivo infection with some of these viruses (LCMV, JUNV) induces high levels of IFN1, ISGs and cytokines suggesting that this strategy is not completely effective or is widely varied in cell type and differs from host to host [134,135,186]. Activation of plasmacytoid DCs (pDC) in response to infection is important for the potent induction of IFN1 and the activation of other immune cell types. It has been shown that non-pathogenic MOPV stimulates a strong pDC response, while pathogenic LASV weakly activates these cells and the response is short-lived [68]. This infers that the impaired pDC activation could be a critical factor in the immunosuppression observed during LASV infection. In the study by Schaeffer and colleagues, MOPV Z protein was detected in pDCs in a pDC /infected Vero E6 co-culture model at a higher level than LASV Z protein [68]. Thus, arenavirus Z proteins may be involved in the activation of pDCs early in infection, although LASV Z proteins appear to be functionally less capable of potently activating IFN1 pathways in pDCs. Conversely, in a study that highlighted the importance of myeloid DC (mDC) activation in the immune response to LASV infection, a LASV/MOPV chimera (in which the MOPV Z protein was swapped into the LASV genome), induced a low level IFN1 response, compared to wild-type LASV infection in an mDC/T cell co-culture model [187]. This suggests that MOPV Z protein, unlike previous findings, is not a modulator of immunogenicity [184,187]. Further, during the development of the measles virus (MeV)-based LASV vaccine candidate, currently being evaluated in phase 1 clinical trials, Mateo and colleagues generated and tested MeV vectors expressing LASV GPC alone or in combination with NP or Z protein for the induction of immune responses in macaques [49]. The authors observed that MeV-Z+GPC vector induced a delayed and diminished IFN1 response in immunised animals, compared to other MeV-LASV vector immunised animals. This lack of IFN1 induction had downstream effects on specific T cell stimulation and on innate and adaptive immune pathways as detected at the transcriptomic level [49]. These findings further imply that in this study, LASV Z is an inefficient activator of IFN1 and T cell responses and provides evidence for the multifaceted role of arenavirus Z proteins in subverting host innate immune pathways.

## 5. Development of NP- and Z-Specific Antivirals

Currently, very limited strategies exist to treat arenavirus infection and rely on early treatment with ribavirin and its analogues, that have very little to no impact on fatality rates [45,188]. In recent years, in addition to the search for effective, preventative vaccines, a number of reports have identified possible protein-specific inhibitors to treat arenavirus infection, but have focused on compounds that interact with the arenavirus glycoproteins and inhibit the entry processes [189,190,191]. Given the multifaceted roles that NP and Z possess to counter host immunity, these proteins could be targeted for the rational design of specific and efficient antiviral therapeutics. Some examples include the treatment of arenaviruses with aliphatic and aromatic disulphide- and azoic-based compounds that target the zinc-binding structure of the Z protein leading to the oxidation of cysteine thiolates and protein aggregation. In these studies against LCMV, JUNV, TCRV and PICV, Garcia and colleagues observed inhibition of virus infectivity and RNA synthesis highlighting the potential of these Z-reactive compounds. Further, these compounds blocked an interaction of Z with the host PML leading to the formation of nuclear bodies and a decrease in virus proliferation [192,193]. In addition, myristic acid analogs were found, in a dose-dependent manner, to inhibit virus production, affecting the localisation of the Z protein and the assembly of JUNV virions, thus targeting protein myristoylation, essential to the function of Z and its involvement in virus propagation [194]. Unlike the Z protein, few NP target compounds have been identified. A potent inhibitor of LCMV replication and budding, KP-146, was identified from a combinatorial library of Krönhke pyridines by Miranda and colleagues, to block the protein-protein interactions of NP and Z; whether these are virus-virus or host-virus interactions remains to be determined [195]. In summary, with the present lack of potential anti-viral drugs and effective vaccines, and the exacerbation of their need by the current LASV outbreaks in West Africa, NP and Z remain important targets for novel therapeutic strategies.

## 6. Conclusions

Arenaviruses utilise host cellular machinery to negotiate host defenses. The remarkably multifunctional viral proteins NP and Z have evolved distinct and synergistic mechanisms to evade the antiviral state induced upon virus infection [110,155,184]. Both proteins are able to counteract RIG-I mediated production of IFN1 thereby inhibiting protein expression and thus dampening innate immune responses; and are able to use their conformational flexibility and protein interaction promiscuity to expand host binding partners to elicit their evasion strategies [132,146,184]. Arenavirus infections in humans with OW and NW viruses can vary from asymptomatic to severe. Importantly, differences in the interactions of NP and Z proteins amongst arenaviruses with the RIG-I, PKR and other immune pathways discussed here, may contribute to this variation in arenavirus pathogenicity and to the molecular determinants of arenavirus virulence. Given the differential immune responses induced particularly by NPs of pathogenic and non-pathogenic arenaviruses, and the lack of direct correlation with disease outcome, it is clear that multiple viral and host genes and the evolution of their protein interactions determines virulence. NP and Z are major contributors to the shape of host antiviral mechanisms. Therefore, developing NP and Z-specific antivirals, coupled with monitoring the evolution of these proteins as the host adapts to their evasive mechanisms, could be used to regulate the control that arenaviruses have on the host immune system, and represents a vital approach to combat these public health and socio-economic burdens [66,196].

## Figures and Tables

**Table 1:** Mechanisms of immune suppression by arenavirus NP exonuclease activity. **√** indicates confirmed interactions/mechanisms. **×** indicates confirmation of no interaction/mechanism reported. n.d. indicates no published data on the interaction. Shaded rows show OW arenaviruses whilst unshaded rows indicate NW arenaviruses. NP, nucleoprotein; IRF3, interferon responsive factor 3; IFN, interferon; IKKε, IκB kinase ε; RIG-I, retinoic acid inducible gene 1; RLR, RIG-I-like receptor; PKR, protein kinase R; MDA-5, melanoma differentiation-associated protein 5; PAMP, pathogen associated molecular pattern; JUNV, Junín virus; MACV, Machupo virus; MOPV, Mopeia virus; LCMV, lymphocytic choriomeningitis virus; LASV, Lassa virus; PICV, Pichinde virus; TCRV, Tacaribe virus.

	dsRNA Binding	dsRNA Degradation	Inhibits Nuclear Translocation of IRF-3 through Interaction with RIG-I and MDA-5	Targets Kinase Domain of IKKε and Blocks Its Activity	Inhibition of PACT-Mediated Augmentation of RIG-I Signalling	Inhibits NF-κB Transcriptional Activation	Activation of PKR	Refs
**Pathogenic**								
LCMV	**√**	n.d.	**√**	**√**	n.d.	**√**	**√**	[102,103,108,116,117,118,119]
LASV	**√**	**√**	**√**	**√**	**√**	**√**	**×**	[96,99,102,103,105,110,115,117,119,120]
JUNV	**×**	**×**	**√**	**√**	**√**	**√**	**√**	[92,102,110,111,114,115,117,119,120,121]
MACV	n.d.	**×**	**√**	n.d.	**√**	**√**	**√**	[102,110,111,115,119,120]
**Non-Pathogenic**								
MOPV	**√**	**√**	n.d.	n.d.	n.d.	n.d.	n.d.	[122]
PICV	**√**	**√**	**√**	**√**	**√**	**√**	n.d.	[102,107,119,120,123]
TCRV	**√**	**√**	**×**	n.d.	**√**	**×**	n.d.	[102,105,119,120]
